# *In vivo* functional neurochemistry of human cortical cholinergic function during visuospatial attention

**DOI:** 10.1371/journal.pone.0171338

**Published:** 2017-02-13

**Authors:** Michael Lindner, Tiffany Bell, Somya Iqbal, Paul Gerald Mullins, Anastasia Christakou

**Affiliations:** 1 Centre for Integrative Neuroscience and Neurodynamics, and School of Psychology and Clinical Language Sciences, University of Reading, Reading, United Kingdom; 2 School of Psychology, Bangor University, Bangor, United Kingdom; National Research Council of Italy, ITALY

## Abstract

Cortical acetylcholine is involved in key cognitive processes such as visuospatial attention. Dysfunction in the cholinergic system has been described in a number of neuropsychiatric disorders. Levels of brain acetylcholine can be pharmacologically manipulated, but it is not possible to directly measure it *in vivo* in humans. However, key parts of its biochemical cascade in neural tissue, such as choline, can be measured using magnetic resonance spectroscopy (MRS). There is evidence that levels of choline may be an indirect but proportional measure of acetylcholine availability in brain tissue. In this study, we measured relative choline levels in the parietal cortex using functional (event-related) MRS (fMRS) during performance of a visuospatial attention task, with a modelling approach verified using simulated data. We describe a task-driven interaction effect on choline concentration, specifically driven by contralateral attention shifts. Our results suggest that choline MRS has the potential to serve as a proxy of brain acetylcholine function in humans.

## Introduction

Cholinergic neurotransmission in the brain is important for cognitive functions such as selective attention [1] and various forms of learning [2], and has been implicated in a number of pathologies, including Alzheimer’s disease [3] and schizophrenia [4]. Cortical acetylcholine (ACH) involvement in visuospatial and sustained attention is well-documented in both animal [5,6] and, indirectly, in human research [7]. Despite its importance, although levels of ACH can be pharmacologically manipulated in both animals and humans, it is not possible to directly measure brain ACH in humans. Therefore, until now, it has not been possible to monitor the unperturbed central cholinergic system in humans. This study was designed to address this gap, using functional magnetic resonance spectroscopy (fMRS).

There is significant evidence for cortical cholinergic involvement in visuospatial attention in the parietal cortex. For example, cholinergic receptors in the parietal cortex of rats are involved in attention processes [8,9], and muscarinic receptor antagonism in humans changes parietal theta oscillations [10,11]. We concentrated on ventral posterior parietal cortex, as there is converging evidence from various techniques for its role in visuospatial attention, including from electrophysiology [12], neuropsychology [13], and neuroimaging [14,15]. Posterior parietal cortex is associated specifically with controlling visuospatial attentional shifts [14,16], while evidence for hemispheric laterality in this involvement has been found using functional magnetic resonance imaging (fMRI) [15], electroencephalography (EEG) [17], and repetitive transcranial magnetic stimulation (rTMS) [18]. Further, systemic administration of the cholinergic agonist physostigmine, leads to a unilateral cholinergic enhancement effect on alpha/beta oscillations in ventral parietal cortex (at the border with occipital cortex; parieto-occipital cortex, or POC) during visuospatial attention shifts, as measured with magnetoencephalography (MEG) [7].

This body of evidence leads to the prediction that cholinergic involvement during shifts of visuospatial attention is unilateral, and happens in the hemisphere opposite the hemispace towards which the shift occurs.

**Fig 1:**
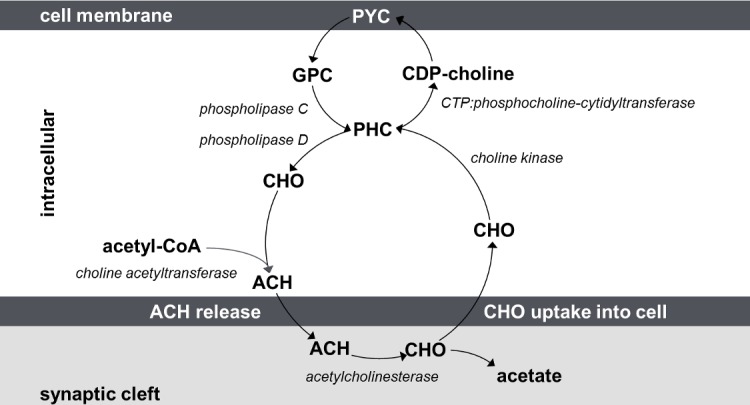
The choline-acetylcholine cycle. The enzyme choline acetyltransferase builds acetylcholine (ACH) from choline (CHO) and acetyl-CoA. After ACH is released in the synaptic cleft, the enzyme acetylcholinesterase converts ACH into the inactive metabolites CHO and acetate. After re-uptake into the pre-synaptic terminal, free CHO is phosphorylated into phosphocholine (PHC), a reaction catalysed by choline kinase. PHC is available to mobilise CHO for further ACH production via phospholipase C. CHO is also bound in the cell membrane as phosphatidylcholine (PYC). PHC can be then converted to CDP-choline by CTP:phosphocholinecytidyltransferase. The enzyme CDP-choline:1,2-diacylglycerol cholinephosphotransferase then converts the CDP-choline into phosphatidylcholine (PYC) [20]. PYC can also be broken down (via phospholipase C) into glycerophosphocholine (GPC), phosphocholine (PHC), and finally CHO (and side products), or (via phospholipase D) directly into free CHO [19]. In the chemical spectra acquired with magnetic resonance spectroscopy (MRS) CHO, GPC and PHC are the only “visible” metabolites of the CHO cycle.

A key part of the ACH biochemical cascade in neural tissue is choline (CHO) (see detailed overview in Fig 1): In neurons, ACH released in the synapse is broken down into CHO and acetate. After CHO is taken up back into the cell, it is immediately phosphorylated into phosphocholine (PHC) by a kinase. CHO is also found bound in the membranes of both neuronal and non-neuronal cells as phosphatidylcholine (PYC). When cell membrane integrity is compromised or altered (e.g. in tumors), PYC can be broken down into glycerophosphocholine (GPC), phosphocholine (PHC), and finally CHO (or directly into free CHO) [19].

Of the metabolites involved in the ACH cycle, CHO, GPC and PHC are “visible” in the chemical spectra acquired with MRS [21,22]. There is evidence that levels of CHO are an indirect but proportional measure of ACH availability in brain tissue [3,21]. It is unlikely that MRS at 3T can resolve the very low free CHO concentration fluctuations that might be directly associated with ACH release [21]. However, it is possible that event-related fluctuations in the cholinergic MRS signal captured at the right timescale could indirectly betray cholinergic contributions to function. For instance, ACH release increases free CHO in the synaptic cleft, as acetylcholinesterase rapidly converts unbound ACH into CHO and acetate. The dynamics of CHO re-uptake into the presynaptic cell are coupled to the rate of ACH release, but the process is slower than ACH release itself [23]. As a consequence, free CHO in the synaptic cleft cannot be transported back into the presynaptic cell instantaneously, imposing a "bottleneck" with increasing CHO concentration in the cleft which may be exploitable. Functional magnetic resonance spectroscopy (fMRS), i.e. the dynamic *in vivo* measure of metabolites (in contrast to average metabolite concentration estimation with MRS), could contribute to this potential application, through tracking putative task-dependent fluctuations in cholinergic metabolites.

Based on this rationale, and on the evidence for cholinergic involvement in visuospatial attention, we measured functional (task-related) cholinergic metabolite levels in the POC using fMRS during performance of a visuospatial attention task. We tested whether fluctuations in CHO concentration, related to cortical ACH release during visuospatial attention, could be captured with MRS. We also used simulated data (with varying ratios of CHO concentrations) to independently confirm the validity of our techniques for the detection of relative task-related changes in CHO. The aim of the study was to provide the first direct evidence for human cortical cholinergic involvement in visuospatial attention shifts. Based on the hemispheric laterality of visual attention [15,17,18] we predicted a specific unilateral increase in CHO during the performance of contralateral attention shifts, a byproduct of increased ACH release into the synapse. A broader aim of the study was to provide direct support for the idea of using cholinergic MRS as a proxy for studying ACH function in the human brain.

## Materials and methods

### Visuospatial attention experiment

#### Participants

Nineteen healthy, right-handed volunteers were recruited from the University of Reading. Due to technical problems three participants had to be excluded from the analysis, because their button presses were not recorded. The remaining 16 participants (11 female) had a mean age of 23.81 years (SD = 5.61). Participants reported no history of neurological disorder and had normal or corrected-to-normal vision. Participants received information about the study in writing, were given the opportunity to ask any questions, and gave written informed consent in advance of their participation. The study was approved by the University of Reading Research Ethics Committee (reference 13/15).

#### Task and procedure

**Fig 2:**
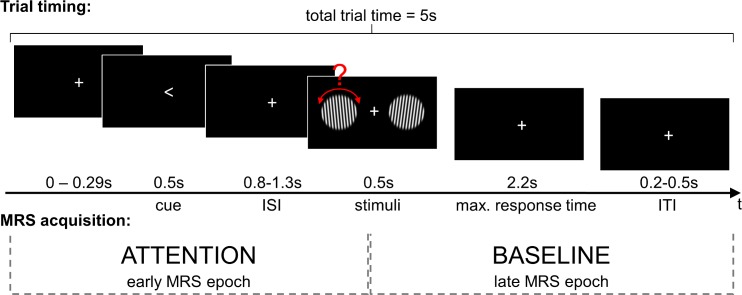
Overview of trial and MRS acquisition timing. Trial timing (top): Each trial had a total length of 5s and begun with a short onset jitter of 0–0.29 s, followed by a cue (0.5s) and a jittered inter stimulus interval (ISI) of 0.8 to 1.3s. The stimuli were presented for 0.5s followed by a maximum response interval of 2.2s and an inter trial interval (ITI) of 0.2 to 0.5s. MRS acquisition (bottom): We collected two MRS acquisitions for each trial (2.5s)—an early epoch, covering the cue, the attention shift phase and part of the stimuli, and a late epoch covering the rest of the trial.

We used a visuospatial attention task which cued participants to covertly shift their attention to one of two spatial locations and report the rotation orientation of a gabor grating stimulus, as described previously [7]. A fixation cross was presented in the centre of the screen during the whole trial. Participants had to fixate it constantly to prevent effects of eye movement in the MRS spectra. In each trial, a 0.5s cue (an arrow pointing left, right, or up) replaced the fixation cross (Fig 2).

The arrow cued participants to shift their attention to the same side of the computer display. Two gabor gratings appeared on the left and right of the display after a jittered interval that lasted between 0.8 and 1.3s. Participants were required to report, through a button press, the tilt orientation (clockwise or counter-clockwise) of the grating that appeared on the cued side, while ignoring the stimulus on the non-cued side of the display.

The discrete experimental conditions were defined depending on whether attention was shifted to the ipsilateral or contralateral hemispace of the display, relative to the voxel placement for MRS acquisition (ipsilateral or contralateral condition respectively). During control trials (neutral condition), the cue was an arrow pointing up, following which participants had to simply press one of the two available buttons. Each trial was split into an early epoch (attention), encompassing the attentional shift, and a late epoch (baseline), during which the response took place. Participants were allowed a maximum of 2.2s to perform their response. The trials ended with a jittered inter-trial interval with a duration between 0.5 and 1s. Each trial had a total duration of 5s (Fig 2).

The position of the MRS voxel (left or right POC) relative to the cue characterized trials as ipsilateral (attention shift in the acquisition hemispace) or contralateral (attention shift in the opposite hemispace). We use the abbreviations “ipsi” and “contra” respectively for the experimental conditions, and “neutral” for the control condition. We used an event-related design with 96 trials in each of three blocks, with a break of 3mins between blocks. The three conditions were counterbalanced over the three blocks and were presented in a pseudo-randomized order, where the same condition was repeated no more than twice in a row.

In advance of data acquisition, participants performed two training sessions inside the scanner (one before the experiment and one during a structural scan) in which the rotation of the gratings (starting with 4°) was adjusted in order to attain an accuracy rate of 90% (over blocks of 24 trials), by increasing (+1°) or decreasing (-0.25°) the degree of rotation. The individual’s rotation degree at the end of each training session was used as the starting degree for the task during the functional localizer and the MRS acquisition (see behavioral results below). During the MRS acquisition the rotation degree was constantly adjusted in the same way, to maintain accuracy at 90%.

We performed two one-way ANOVAs for repeated-measures to assess any differences in reaction time (RT) or accuracy between the ipsilateral, contralateral and neutral conditions.

#### Data acquisition

All ^1^H-MRS spectra and MR images were acquired on a Siemens TRIO MRI System (Centre for Integrative Neuroscience and Neurodynamics, Reading, UK) using an eight channel head coil.

#### Structural scan

Following the functional localizer, we obtained a high resolution whole brain T1 weighted image (1x1x1mm voxel resolution, field of view = 250mm, 176 slices, TR = 2020ms, TE = 2.9ms, flip angle = 9°). Both the functional localizer and the structural scan were used to guide the individual anatomical localization for the MRS voxel placement.

#### fMRI localizer for MRS voxel placement

**Fig 3:**
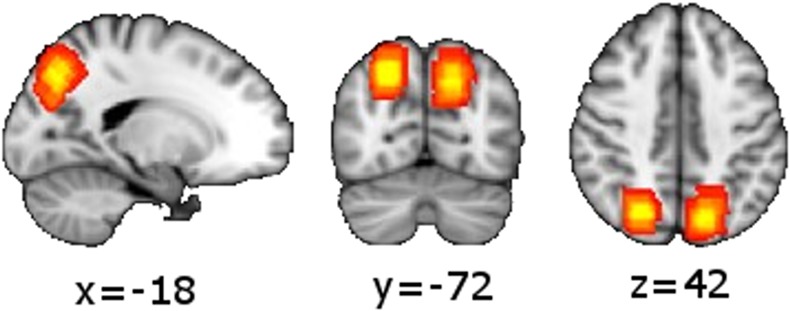
Sum of MRS voxels over all subjects. For better visualization the MRS voxel masks were transformed to MNI space. All subjects contributed to coordinates with the highest overlap, including the average center coordinate (MNI -18, -72, 42) (yellow color).

For the functional magnetic resonance imaging (fMRI) localizer (3x3x3mm voxel resolution, field of view = 192mm, 27 slices, TR = 2000ms, TE = 32ms, flip angle = 90°) we used a block design with 12 trials per block (either only control trials or a mixture of experimental (i.e. attention shift) trials) and 3 blocks of each type. We used the online General Linear Model analysis software in the SIEMENS echo planar imaging sequence to contrast experimental vs. control conditions, identifying participant-specific functional activation in the POC during visuospatial attention shifts compared to the control condition. Individual activation in the POC was used as the centre for the MRS voxel positioning. See Fig 3 for the sum of the voxel position maps, i.e. the voxel position overlap, in MNI space.

#### MRS single voxel event-related acquisition

For each participant three blocks of 192 single voxel point-resolved spectroscopy sequence (PRESS) acquisitions (15x15x15mm, TR = 2500ms, TE = 30ms, no averages, advanced shim mode) were acquired in the POC (for 7 participants in the left hemisphere and for 9 in the right). For each of the experimental trials (N = 288) two single voxel ^1^H-MRS measurements were acquired–an early and a late epoch. The onsets of the trials were jittered (between 0 and 290ms; mean = 108ms, SD = 66) in relation to the onset of the early epoch (see Fig 2). The trial and acquisition timings ensured that attention shifts occurred only during the first epoch. After each of the three blocks, corresponding spectra without water suppression (15x15x15mm, TR = 2000ms, TE = 30ms, 16 averages, advanced shim mode)at the same voxel position were acquired as reference.

Because it is not possible to implement post-acquisition movement correction in MRS, we used the SIEMENS Auto Align Scout for all measures to minimize the variability of the voxel position in the three MRS sequences by adjusting the voxel position to the actual head position of the participant before each MRS sequence.

#### MRS data analysis

Preprocessing and data analyses were performed using java-based Magnetic Resonance User Interface (jMRUI; software version 4.0 (http://www.mrui.uab.es/mrui) [24]).

Spectra from incorrect trials were removed, resulting in between 65 to100 (mean: 85.92 measures, SD = 8.75) measures per condition. The spectra from each single volume scan in a sequence were phase-corrected using the subsequent H_2_O reference scan. We averaged spectra of ipsilateral, contralateral and neutral trials for the early (attention) and the late epochs (baseline) separately for right and left hemisphere voxels. The averages were then pre-processed in JMRUI (removing the water peak and apodizing with a 3Hz Gaussian kernel). The non-linear least squares fitting algorithm (Automated Quantification of Short Echo time MRS Signals, AQSES, [25]) within jMRUI was used to estimate metabolite concentrations. We used a simulated model basis sets for 17 metabolites (acetate, aspartate, CHO, creatine, gamma-aminobutyric acid, glucose, glutamate (GLU), glutamine, GPC, lactate, myo-inositol, N-acetylaspartate (NAA), phosphocreatine, PHC, scyllo-inositol, succinate, and taurine).

**Fig 4:**
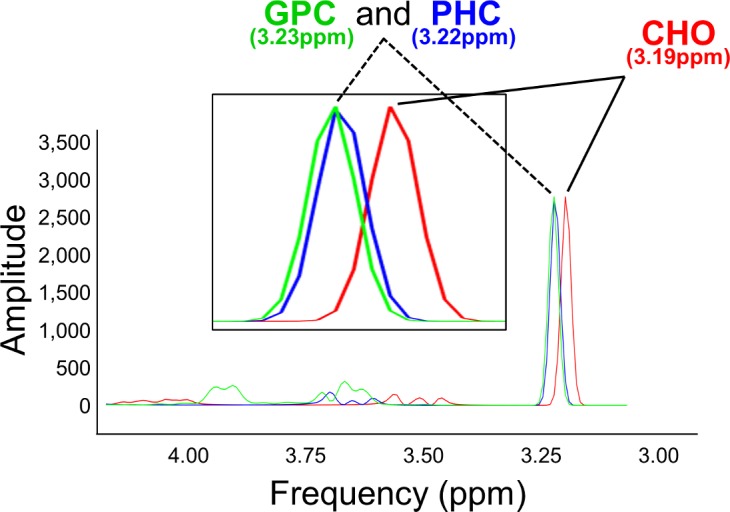
Choline-containing metabolite spectrum. Spectral patterns for the metabolites CHO, PHC and GPC simulated using VeSPA (https://scion.duhs.duke.edu/vespa/project). Metabolites were simulated at a field strength of 3T (main field 123.25MHz) using a PRESS pulse sequence (TE1 = 20ms, TE2 = 10ms).

The detection of CHO changes separately from changes in other choline-containing metabolites (CCMs) is not trivial in MRS using a 3T MRI scanner. The detectable CCM peaks are very close to each other (Fig 4). Consequently, typical MRS studies use models for quantitation of global concentration of cholinergic metabolites [26]. In our study, because of the strong biochemical hypothesis, we are especially interested in the relative differences in CHO concentration between task conditions separately from other CCMs.

Therefore, we modeled CHO separately from a PHC/GPC combined peak (model MOD_CHO_sep_). It is important to demonstrate that this strategy is robust and serves the experimental question. We therefore also implemented the global CCM modelling strategy, by also using a model where all 3 CCMs were modelled together into one peak (model MOD_CHO_global_).

For both models we used a previously described optimization procedure for model fitting [27]: The NAA peak of the standard metabolite model was aligned to 2.02ppm. The averaged data for each condition for each participant was also aligned so that the NAA peak was at 2.02ppm, as was the NAA peak of the averaged data of all conditions for each participant. The model was run in AQSES with equal phase for all metabolites (begin time fixed; delta damping: -10 to 25Hz; delta frequency: -5 to 5Hz; no background handling; 0 truncated points; 2048 points; normalization: on).

We performed a partial volume correction to account for individual voxel tissue concentration based on a procedure described in [28]. The high-resolution T1 weighted images were segmented into grey and white matter using Statistical Parametric Mapping toolbox (SPM), so tissue content within the MRS voxels could be assessed [29]. Voxel registration was performed using custom-made scripts developed in MATLAB (Release 2012b, The MathWorks, Inc., Natick, MA, USA), generating a mask for voxel location by combining location information from the Siemens raw file with orientation and location information contained within the T1 image. The application of this mask to the grey matter, white matter and cerebrospinal fluid (CSF) images enabled the calculation of partial volume within the region of interest by establishing the percentage of each tissue type within the relevant voxels. These percentages were used to correct metabolite concentrations for partial volume and relaxation effects as described by [29].

To exclude potential blood-oxygen-level-dependent related effects on any concentration levels reported and possible water changes during neuronal activity, the results were referenced to another metabolite. Typically, NAA, creatine (CRE) or water are used as reference metabolites in MRS [30]. The estimated concentrations of CRE or NAA did not show task related concentration changes (see Results section for details). Because we optimized the detection of our spectra by maximizing the NAA peak, we did not use NAA as reference to avoid any direct effect of the optimization on the referenced CHO concentration values. CRE did not show any task related effects or interactions. Therefore, we used CRE as reference for our metabolites of interest (MOIs).

Tests of significance were performed using two-factorial ANOVAs for repeated measures with the factors condition (ipsilateral, neutral and contralateral) and epoch (attention vs. baseline) controlling for mean reaction time in the three conditions. We performed this test for all MOIs.

We also checked for effects of potential nuisance variables such as gender, age and time-of-day of the scans. We used the Bonferroni method to correct for multiple comparisons.

### Simulation

#### Data simulation

We simulated metabolite peaks using the simulation option "NMR-Scope" of jMRUI [24] to produce synthetic data equivalent to data acquired at 3T. We used the same parameters as in our experiment: TE = 30ms, 2048 sampling points. We simulated 17 metabolites with amounts derived from independent experimental testing (acetate, aspartate, CHO, creatine, gamma-aminobutyric acid, glucose, glutamate, glutamine, GPC, lactate, myo-inositol, N-acetylaspartate (NAA), phosphocreatine, PHC, scyllo-inositol, succinate, taurine). We simulated all combinations of 11 different amounts of CHO and 11 different amounts of PHC (the range for both was from 0 (no CHO/PHC at all) to 2/3 of the NAA peak. We then added the same amount of white noise to this data as we had in our real data (mean SD~ = 200).

For all combinations of simulated CHO and PHC we created 20 iterations using MATLAB, which were imported into jMRUI for analysis.

#### Data analysis

Data analyses were performed using jMRUI [24] with the quantitation procedure AQSES as we did with the experimental data. We analyzed all 20 iterations with each model (MOD_CHO_global_ and MOD_CHO_sep_). In order to test whether we could discriminate between metabolite levels, we performed Mann-Whitney-Wilcoxon (MWW) tests between the detected amplitude of neighboring levels of each CHO and PHC. The results were corrected for multiple comparisons using false discovery rate correction (FDR; q<0.05).

For visualization, we averaged the 20 iterations of each combination and analyzed the average datasets with both the MOD_CHO_sep_ and MOD_CHO_global_ models.

## Results

Using fMRS, we detected a task-related increase in CHO following visuospatial attention shifts. Importantly, this effect was specific to CHO, and specific to the MRS acquisition voxel contralateral to the attentional shift, in line with predictions.

### Visuospatial attention experiment

#### Behavioral data

The individual detectable rotation degrees at the beginning of the MRS sessions (starting degree) ranged from 0.76° to 4.25° (mean = 1.82°, SE = 0.27). The mean degree during the MRS sessions ranged from 0.70° to 4.95° (mean = 1.95°, SE = 0.32). The initial and mean degree did not correlate with participant gender or age, nor with the time of the day that the testing took place. The average reaction time (RT) for the neutral condition (327ms, SE = 23.0) was significantly faster than for the two other conditions (ipsilateral: 605ms, SE = 36.1; contralateral: 622 ms, SE = 39.0), driving a significant main effect of conditions (F(2,14) = 56.952, p <<0.05). There was a small but statistically significant difference in RT between the ipsilateral and contralateral conditions (t(15) = -2.320, p<0.05), however RTs were highly correlated between the two conditions over participants (r(14) = 0.982, p<0.001). Nevertheless, we controlled for RT in the MRS data analyses. The two experimental conditions (ipsilateral and contralateral) did not differ in accuracy (t(15) = 1.324, p = 0.205).

#### MRS data: Effects of interest

**Fig 5:**
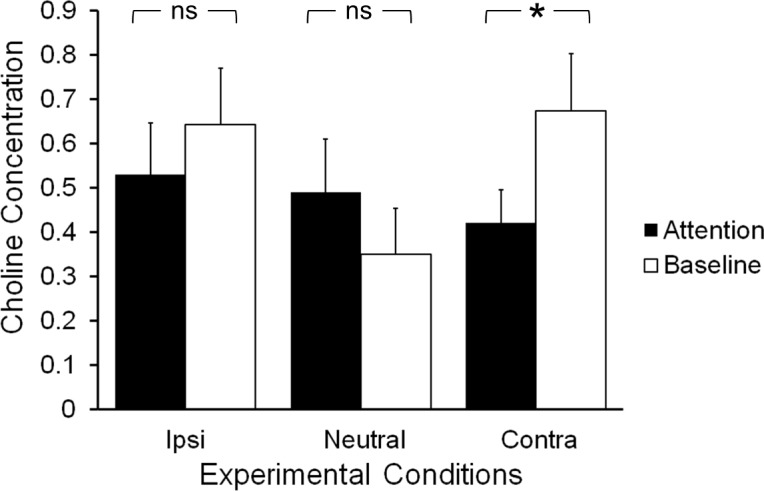
Task-related choline changes. Changes in the concentration of choline between the early and late epochs (early-late) in the different experimental conditions (Ipsi: MRS acquisition ipsilateral to the shift; Neutral: no attention shift (free choice) trial; Contra: MRS acquisition contralateral to the attention shift).Choline increased only after contralateral visuospatial attention shifts, and there was a statistically significant interaction between condition and epoch. Error bars indicate the standard error.

CHO levels were increased following the attention epoch during baseline, but only in the contralateral condition (see Fig 5). An analysis of the CHO concentrations using a two-factorial ANOVA for repeated measures, controlling for RT, revealed a significant interaction between condition and epoch (F(2) = 5.433, p<0.05, partial η^2^ = 0.312), but no significant main effects of condition (F(2) = 0.081, p = 0.922) or epoch (F(2) = 1.976, p = 0.185). Paired samples t-tests of the change in concentration between epochs showed that the change was significant in the contralateral condition (t(15) = -2.124, p = 0.051), but not in the ipsilateral (t(15) = -0.754, p = 0.462) or neutral conditions (t(15) = 1.335, p = 0.202).

This effect was specific to CHO (as quantified with MOD_CHO_sep_). The combined PHC/GPC concentration estimate (quantified with the same model) showed no main effect of condition (F(2) = 0.395, p = 0.678) or epoch (F(2) = 0.668, p = 0.430), and no condition by epoch interaction (F(2) = 2.437, p = 0.109). Similarly, the combined choline-containing compounds concentration estimate (quantified with MOD_CHO_global_) showed no main effect of condition (F(2) = 1.326, p = 0.284) or epoch (F(2) = 0.334, p = 0.574), and no condition by epoch interaction (F(2) = 1.870, p = 0.176).

#### MRS data: Acquisition, functional, and nuisance variable controls

Acquisition hemisphere; effects on CHO concentration: There was no main effect of hemisphere as a between-subjects factor (F(1) = 0.148, p = 0.708) on the repeated measures ANCOVA (controlling for RT) of CHO levels across condition (ipsilateral, contralateral and control) and trial epoch (attention versus baseline), and no 3-way interaction (F(2) = 1.134, p = 0.340), but there remained a significant condition by trial epoch interaction (F(2) = 6.170, p = 0.007), as in the effect of interest (above).

Acquisition hemisphere; effects on variance of CHO concentration estimate: Cramer Rao lower bounds were low for the estimates of all metabolites of interest (less than 30% of metabolite concentration) [31]. There were no effects of acquisition hemisphere on CHO CRB levels, regardless of condition and trial epoch (all t-tests: p>0.5).

Acquisition hemisphere; effects on signal-to-noise ratio: The signal-to-noise ratio (SNR) was quantified as the average CRE signal divided by the average noise (amplitude variance outside the metabolite region) in the control condition. Acquisition hemisphere had no effect on SNR (L: 1.018 (0.878), R: 0.881 (0.476); t(14) = 0.402, p = 0.694).

Acquisition hemisphere; effects on tissue composition: There was no effect of hemisphere on the tissue composition of the acquired voxels as shown by independent samples t-tests (left versus right) for grey matter (t(14) = 0.855, p = 0.407), white matter (t(14) = -0.563, p = 0.582), and cerebrospinal fluid (t(14) = -0.653, p = 0.524).

**Fig 6:**
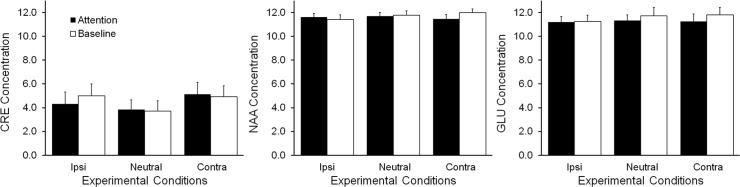
Reference metabolites and functional control. There were no effects of condition (ipsilateral, neutral or contralateral) or epoch (attention or baseline), and no condition by epoch interaction on reference and control metabolites: CRE (left), NAA (middle) and GLU (right).

Reference metabolites: To show that the effects of interest were not global effects of the measurement procedure, we checked for task-related effects on CRE and NAA (Fig 6, unreferenced values, left and middle panels respectively). There was no main effect of condition (F(2) = 1.327, p = 0.280) or epoch (F(2) = 0.043, p = 0.838) on CRE, and no condition by epoch interaction (F(2) = 0.387, p = 0.683). Similarly, there was no main effect of condition (F(2) = 0.626, p = 0.541) or epoch (F(2) = 1.074, p = 0.316) on NAA, and no condition by epoch interaction (F(2) = 2.673, p = 0.085).

Functional metabolite control: Glutamate (GLU) release is likely to accompany attentional shifts, given observed BOLD changes in similar tasks [16]. However, if GLU involvement could be measured with fMRS, we had no reason to expect it to be unilateral, in contrast to our main hypothesis about CHO involvement. Glutamate (GLU) is detectable with the point-resolved spectroscopy (PRESS) sequence that we used for data acquisition [32]. However, there was no effect of condition (F(2) = 0.198, p = 0.821) or epoch (F(1) = 0.596, p = 0.452), and no condition by epoch interaction (F(2) = 0.078, p = 0.925) on GLU concentration (Fig 6).

Nuisance variable controls: Age, voxel tissue composition (i.e. percentage of white matter, grey matter or CSF) and testing time-of-day did not show any correlations with the concentrations of the MOIs (CHO, GLU; NAA and CRE) during the two trial epochs (all Pearson’s correlation tests, corrected for multiple comparisons, p>0.05). There was also no effect of gender on any metabolite concentrations (all independent samples t-tests (males versus females): p>0.05), with the exception of GLU concentration during the baseline epoch of the neutral condition (t(14) = -3.685, p = 0.002), which nonetheless did not alter the reported effects.

### Simulation

To independently compare our model (MOD_CHO_sep_) with the alternative model (MOD_CHO_global_) regarding their ability to detect relative changes in CHO, we created simulated MRS datasets with different amounts of CHO and PHC (see methods section for more details).

**Fig 7:**
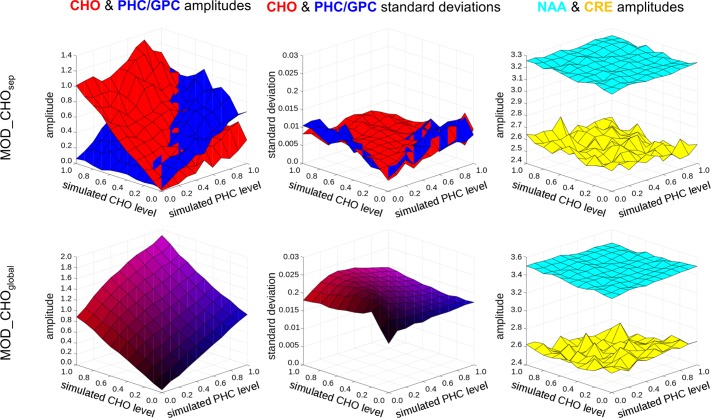
Choline-containing metabolite amplitude changes in synthetic data. Amplitudes of choline (CHO, red) and the combined phosphocholine/glycerophosphocholine peak (PHC/GPC, blue) estimated using the MOD_CHOsep (top) and MOD_CHOglobal (bottom) models in synthetic data. Estimated levels of CHO (red) and PHC/GPC (blue) show an increase in line with the simulated levels of CHO and PHC. Also shown are the levels of NAA and CRE and GPC (right panel). Note that the bottom panel shows combined amplitudes for choline-containing compounds as the MOD_CHOglobal model does not differentiate between CHO and other choline-containing metabolites.

We used simulated data to test the possibility of detecting relative changes in cholinergic metabolites in different data sets. Because we were especially interested in the relative changes in CHO, we simulated data with different levels of CHO and PHC while keeping all other metabolites constant. Fig 7 shows the detected amplitudes of CHO, PHC/GPC, NAA and CRE from the analysis of the averaged simulated data using both models.

The quantitation of the simulated data averaged over iterations using MOD_CHO_sep_ shows relative increases in the CHO and PHC levels which are fairly independent from each other, providing evidence that the model can discriminate the metabolites at 3T-relevant, physiological concentrations and noise levels. The standard deviations of the amplitudes are stable and small for all CHO and PHC combinations. The NAA, CRE and GPC amplitudes are simulated to be stable over all CHO and PHC combinations, so they control for systematic error in the process. The total amount of detected cholinergic metabolites is higher using the MOD_CHO_global_. However, the standard deviation increases with increasing CHO and PHC levels than in the MOD_CHO_sep_ analysis.

**Fig 8:**
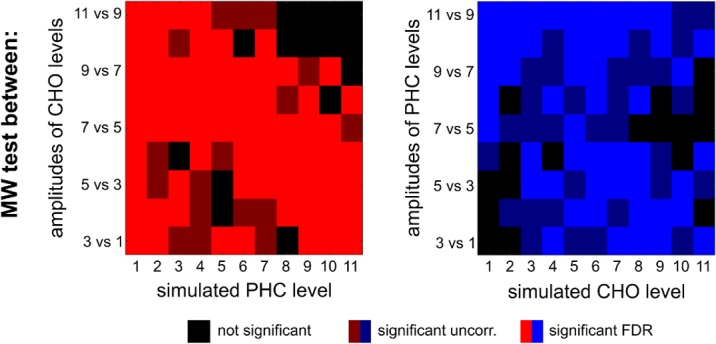
Sensitivity of amplitude change detection. Results of the significance tests between neighboring CHO (red) and PHC (blue) simulated levels over 20 iterations. Bright colors represent differences between the neighboring levels detected as statistically significant after correcting for multiple comparison using FDR (q<0.05). Dark colors represent uncorrected significance and black denotes no significant result.

MWW tests revealed significant increase between neighboring CHO levels in 82.8% of cases (67.7% after correction for multiple comparison using FDR). For PHC, this rate was 82.8% (50.5% after FDR) (Fig 8).

## Discussion

This study provides direct evidence for the involvement of the human cortical cholinergic system in visuospatial attention. We used event-related MRS to track changes in the concentration of CHO in the POC during visuospatial attention shifts. We show task-related changes in CHO driven by concentration increases after attention shifts in the contralateral, but not in the ipsilateral hemi-field. This finding is in line with prior evidence for cholinergic involvement in visuospatial attention in this region [7], and for the laterality of processing attentional shifts [15,17,18]. This laterality effect is thought to be related to an indirect unilateral coupling of ACH release from the prefrontal to the parietal cortex in attention processes [33].

The average CHO concentration in brain tissue shows age related changes [34], and fluctuates with time of day because of different reasons, e.g. alcohol usage [35], athletic skills [36], and dietary CHO uptake [37]. Despite such possible confounding effects, we show that the task specific changes in CHO concentration do not correlate with the age of the participants, nor with the time-of-day of the scans.

The frequencies of the choline containing metabolites are very close in the spectra acquired with MRS at 3T, and it may be thought that fitting CHO separately from other CHO-containing peaks is problematic given that the resulting concentration estimates are anti-correlated because of their interdependence. This is a valid source of skepticism, and significant further development is needed to fully validate the cholinergic fMRS paradigm. However, in this study we explicitly address the modelling challenges using extensive simulation data. Although the dynamics of CHO and PHC (in particular) are coupled in the ACH cycle (Fig 1), there are physiological reasons why this coupling is non-linear as outlined above, which can be capitalized to further disentangle functional from model-fitting effects.

Importantly, we found a robust effect for CHO, but not for PHC and GPC (the other contributors to the cholinergic MRS footprint), not for the choline-containing metabolites fitted together, and not for GLU (our non-cholinergic functional control). This effect was specific to the contralateral condition, compared to both the intra-subject ipsilateral and control conditions, in line with our theoretical prediction.

In addition, using simulated data, we demonstrated that it is possible to detect relative differences in CHO levels using our model, which showed an accurate fit. It has been demonstrated that the composite cholinergic ^1^H MRS peak at 3T correlates best with CHO and to a lesser extent with PHC [21]. Therefore, even if absolute CHO quantification were not possible at 3T, our findings suggest it may be better to model CHO separately to decrease the overall error in fitting. This captures more of the variability of the cholinergic metabolite peak complexes (which are not single peaks) and of their relationship to each other. The relative changes in CHO and the combined PCH and GPC estimate measured between different spectra were very accurate, allowing relative changes in CHO during different epochs of the task to be robustly detected. Therefore, when MRS is used for functional (task-related) measurement of CHO, our model can be used within-subjects to detect the relative differences in CHO levels between conditions.

A key question for further validation of cholinergic MRS to study human ACH function will be to establish the conditions under which phasic concentration changes in choline containing metabolites may be quantifiable with MRS. For the time being, we postulate that, in our experiment, the task-dependent changes in CHO concentration were driven by increases in extracellular CHO concentration following ACH release during the attentional shift, as acetylcholinesterase converts unbound ACH into CHO and acetate. Animal experiments show that extracellular choline levels can increase more than three-fold following ACH hydrolysis (although this process may not be contained to the synapse) [38].

ACH hydrolysis is followed by the reuptake of CHO into to presynaptic cell via the high affinity choline transporter (CHT), a sodium- and chloride-dependent protein expressed selectively in cholinergic neurons [39,40]. CHO transport is thought to be the rate-limiting regulatory step in ACH synthesis, providing a direct link between CHO and ACH concentration dynamics [23,41]. CHT is tightly regulated with respect to cholinergic signaling through its steady state-enrichment on terminal vesicles and activity-dependent shuttling to the plasma membrane in response to presynaptic excitation [42–45]. As a consequence, the prospect of imaging activity-dependent cholinergic regulation is significantly enhanced by recent efforts to characterize it as a potential pharmacological intervention target [42]. Importantly, there is recent evidence that a genetic polymorphism of CHT, which limits CHO transport capacity, is associated with decreased recruitment of dorsolateral prefrontal cortex under increased attentional demands [46].

Our current conceptualization of this mechanism is further supported by evidence that increased extracellular CHO may further down-regulate its re-uptake mechanism [47], possibly by inducing a rapid decrease in cell-surface expression of its high affinity transporter CHT1 [41]. Therefore, individuals for whom the balance of ACH break-down in the synapse and CHO re-uptake is not efficient, would show progressively increasing CHO and, ultimately, potentially compromised ACH function. This hypothesis can be directly tested in an appropriate animal model and with human MRS.

This rationale is in line with evidence that attentional enhancement following systemic physostigmine administration is due to cumulative effects, asynchronous with the shifting process. Physostigmine inhibits acetylcholinesterase, and therefore acts via modulation of ACH break-down in the synapse, as opposed to ACH release. For example, in a systemic cholinergic enhancement study the maximum enhancement effect on spatial attention (in a task similar to the one used here) was found in the post-stimulus phase, and not during shifting [7], supporting the idea that the rate of CHO re-uptake and phosphorylation may be driving the observed modulatory effect. In other words, physostigmine administration may act to alleviate inefficiency, rather than enhance function per se.

The results of this study suggest that relative CHO levels, as measured with event-related MRS, may be associated with the well-established function of cortical ACH in visuospatial attention shifts. This is in line with evidence from animal models and slice preparation studies that demonstrates a relationship between tissue availability of CHO and ACH concentration [3,48].

The open questions outlined above notwithstanding, our findings support the idea that cholinergic MRS can be a useful proxy for the study of ACH function, a prospect which warrants further attention. Importantly, our data suggests that relative functional fluctuations between CHO and other MRS-visible cholinergic metabolites can be distinguished at 3T. This opens up the possibility of tractable broader opportunities for *in vivo* functional neurochemical imaging of the human cholinergic system, which is currently not part of the empirical agenda.

Our findings more generally suggest that event-related measurement of brain metabolites with fMRS may allow for characterization of *in vivo* functional neurochemistry under conditions with strong a priori hypothesized involvement, and thus support previous studies investigating other neurometabolites [49–57].

In summary, we describe a task-driven increase in CHO in the parietal cortex, specifically following contralateral attention shifts. To our knowledge, this is the first time that event-related MRS has been used to detect functional changes in the human central cholinergic system associated with attention. Further refinement of *in vivo* functional measurement of the cholinergic system could prove invaluable in the early diagnosis and treatment of diseases that affect cholinergic dysfunction (such as Alzheimer's disease), as well as in charting the progression of non-pathological age-related cognitive decline.
